# Insufflation in Intestinal Obstruction

**Published:** 1875-10

**Authors:** 


Insufflation in Intestinal Obstruction.—Dr. Brun, in a communication to the *Journal de Medicine* of last month, describes a case of intestinal obstruction, due to the ingestion of large quantities of service fruit, which was successfully relieved by means of J. Wood's method of insufflation of air. The difficulty had lasted four days, and had resisted all the ordinary methods of treatment. Moreover, the procedure recommended by Taliaferra of injecting into the bowels bicarbonate of soda and citric acid separately, for the purpose of liberating gas in the intestines and distending the canal, had been tried ineffectually. The patient was evidently sinking; the pulse was very feeble, and there was stercoraceous vomiting. An œsophagal sound was introduced into the rectum, and to the external extremity the nozzle of an ordinary bellows was attached. Air was then injected till the abdomen had increased markedly in volume. The first attempt, however, was unsuccessful. It was repeated, and the insufflation continued until considerable abdominal tension was produced, and respiration was rendered difficult. In the course of an hour dejections began and the patient recovered.—*Medical Record.*

